# O-1602, an Agonist of Atypical Cannabinoid Receptors GPR55, Reverses the Symptoms of Depression and Detrusor Overactivity in Rats Subjected to Corticosterone Treatment

**DOI:** 10.3389/fphar.2020.01002

**Published:** 2020-07-08

**Authors:** Andrzej Wróbel, Anna Serefko, Aleksandra Szopa, Daniela Ulrich, Ewa Poleszak, Tomasz Rechberger

**Affiliations:** ^1^Second Department of Gynecology, Medical University of Lublin, Lublin, Poland; ^2^Laboratory of Preclinical Testing, Chair and Department of Applied and Social Pharmacy, Medical University of Lublin, Lublin, Poland; ^3^Department of Obstetrics and Gynaecology, Medical University Graz, Graz, Germany

**Keywords:** depression, detrusor overactivity, receptor GPR55, O-1602, rats

## Abstract

In view of the fact that GPR55 receptors are localized in brain areas implicated in the pathophysiology of depression, GPR55 gene expression is reduced in the dorsolateral prefrontal cortex of suicide victims, and GPR55 receptor agonism exerts an anxiolytic-like effect, GPR55 receptors have drawn our attention as a potential target in the treatment of mood disorders. Therefore, in the present study, we wanted to check whether a 7-day intravenous administration of O-1602 (0.25 mg/kg/day) – a phytocannabinoid-like analogue of cannabidiol that belongs to the agonists of GPR55 receptors, was able to reverse the corticosterone-induced depressive-like behavior accompanied by detrusor overactivity in female Wistar rats. Additionally, we tried to determine the influence of GPR55 stimulation on the bladder, hippocampal and urine levels of several biomarkers that play a role in the functioning of the urinary bladder and/or the pathophysiology of depression. Our experiments showed that O-1602 therapy improved signs of depression (measured by the forced swim test) and detrusor contractility (measured by conscious cystometry) in animals exposed to the corticosterone treatment. Moreover, the treatment reduced the oxidative damage in the urinary bladder and neuroinflammation (observed as the reduction of elevated levels of 3-NIT, MAL, and IL-1β, TNF-α, CRF, respectively). The O-1602 treatment also reversed the abnormal changes in the bladder, hippocampal or urine values of CGRP, OCT3, VAChT, BDNF, and NGF. The above-mentioned findings allow to suggest that in the future the modulation of atypical cannabinoid receptors GPR55 could have a potential role in the treatment of depression and overactive bladder.

## Introduction

The endocannabinoid system as well as both exogenous and endogenous ligands of the cannabinoid receptors have attracted scientists’ attention for decades. Apart from their noxious psychoactive properties, cannabinoids exert a variety of positive biological effects. They could be promising analgesic, anti-inflammatory, anti-allergic, anti-emetic, antineoplastic, muscle relaxant, immunosuppressive, bronchodilator, sedative, and neuroprotective agents. Additionally, they can also improve mood, reduce the intraocular pressure, and stimulate appetite. Generally, both endogenous and exogenous cannabinoids act *via* 2 typical G-protein coupled cannabinoid receptors – CB_1_ and CB_2_, which can be found in the periphery and in the brain (Pertwee, 2015). However, quite recently, it has been demonstrated that some of the biological effects of cannabinoids are CB_1_/CB_2_ receptor-independent and the existence of so-called atypical cannabinoid receptors has been discovered. Amongst them, the orphan metabotropic receptor GPR55, sometimes referred to as the CB_3_ receptor, is mentioned (Alavi et al., 2018). GPR55 receptors have a low sequence homology to CB_1_ (13.5%) and CB_2_ (14.4%) receptors (Pertwee, 2007). They are modulated by several diverse non-cannabinoid (i.e., L-lipophosphatidylinostiol – an endogenous lipid mediator) and cannabinoid ligands, including: endogenous cannabnoids – anandamide and 2-arachidonoylglycerol, phytocannabinoid – delta-9-tetrahydrocannabinol, synthetic cannabinoids – JWH-015, rimonabant, AM251, and atypical cannabinoid – O-1602 (Pertwee, 2007; Alavi et al., 2018). It has been revealed that GPR55 receptors activate G_q_ and G_α12/13_ proteins and affect different signaling pathways like calcium release from the intracellular stores and the signaling dependent on Rho kinase, small GTPases (RhoA, cdc42, rac1), nuclear factor kappa-light-chain-enhancer of activated B cells (NF-κB), nuclear factor of activated T-cells (NFAT), and cAMP response element binding (CREB) (Baker et al., 2006). GPR55 receptors are found in numerous cells and organs, such as the osteoclasts, cancer cells, liver, adrenal glands, spleen, small intestine, pancreas, lungs, and kidneys. Their activity is associated with mechanisms of bone formation, glucose homeostasis, inflammatory response, neuropathic pain, angiogenesis, fetoplacental development, oncogenesis, and others (Alavi et al., 2018). Existence of GPR55 receptors has also been detected in different parts of the brain, including the striatum, hippocampus, forebrain, cortex, and cerebellum (Wu et al., 2013). As a consequence, involvement of these receptors in hyperlagesia (Staton et al., 2008), pain perception (Deliu et al., 2015), motor coordination (Wu et al., 2013), anxiety (Rahimi et al., 2015; Shi et al., 2017), substance abuse, and neuroprotection has been demonstrated (Alavi et al., 2016; Hill et al., 2019). Most probably, GPR55 receptors also participate in the hippocampal plasticity, and may play an important role in the modulation of memory and learning (Hurst et al., 2017) as well as in the control of decision-making (Garcia-Gutierrez et al., 2018). Wu et al. (2013) reported that mice with GPR55 knock-out did not present any significant brain defects, including potential disturbances in the endocannabinoid system. Apart from some deficits in motor coordination and thermal sensitivity, animals deprived of GPR55 receptors behaved similarly to their wild-type counterparts in the recognized tests measuring anxiety, depression, sensory-motor gating, fear conditioning, gross motor skills, and muscle strength.

Bearing in mind the following facts: (1) localization of GPR55 receptors in the brain areas implicated in the pathophysiology of depression (Wu et al., 2013), (2) reduced GPR55 gene expression in the dorsolateral prefrontal cortex of suicide victims (Garcia-Gutierrez et al., 2018), and (3) anxiolytic-like effect of GPR55 receptor agonism (Rahimi et al., 2015; Shi et al., 2017), GPR55 receptors have drawn our attention as a potential target in the treatment of mood disorders. Depression, as a disease that affects over 322 million people worldwide, poses a major global health issue. Its treatment is highly complicated since the currently available antidepressant drugs are not sufficiently effective, they have a delayed onset of biological action and a high potential to induce adverse reactions (Cipriani et al., 2018). Therefore, new therapeutic solutions in the treatment of depressive disorders are needed. Stimulation of GPR55 receptors could be one of them – that is why in the present study we wanted to find out whether administration of O-1602 (a phytocannabinoid-like analogue of cannabidiol that belongs to the agonists of GPR55 receptors) was able to reverse the corticosterone-induced depressive-like behaviour accompanied by detrusor overactivity in rats. Importantly, atypical cannabinoids not binding to CB_1_ and CB_2_ receptors do not exert psychotropic actions and potentially could be a safer alternative to the classical ones (Schuelert and McDougall, 2011). In our experiments, we decided to use a bi-directional animal model since bladder dysfunctions (including bladder overactivity) frequently co-exist with depression. Similarly to depression, treatment of overactive bladder is also highly problematic. Anticholinergic drugs used as the first-line therapy for this disease act gradually, may induce bothersome adverse effects, and not all patients respond to these agents (Leron et al., 2018). According to the literature data (Hirayama et al., 2012), depression belongs to the independent risk factors for overactive bladder and patients with bladder overactivity are more prone to develop depression (Stewart et al., 2003). The association between depressive disorders and overactive bladder is explained by the participation of the same signalling pathways in the pathophysiology of both diseases, such as the hypothalamic-pituitary-adrenal axis, serotoninergic neurotransmission, inflammatory processes) (Ribeiro Melotti et al., 2018), or the endocannabinoid system. It has been shown that endocannabinoids regulate micturition and bladder functions, and most probably, they are implicated in the pathogenesis of detrusor overactivity (Bakali et al., 2016).

Therefore, we presumed that activation of GPR55 receptors could be an effective strategy in both above-mentioned diseases. The outcomes of our previous projects confirmed that a 14-day exposure to corticosterone generates both depressive phenotype and symptoms of detrusor overactivity in female rats and that these effects are devoid of histopathological damages in the urinary bladder (Wrobel et al., 2016). Moreover, the corticosterone model makes possible to assess signs of inflammation and disturbances in concentrations of neurotrophic factors, which are specific to depression and detrusor dysfunctions (Wrobel et al., 2018).

In the present study, we also tried to determine the influence of GPR55 stimulation on the levels of the following parameters in the bladder urothelium: calcitonin gene related peptide (i.e., a neuropeptide involved in nociception, CGRP), malondialdehyde (i.e., a marker of lipid peroxidation, MAL), 3-nitrotyrosine (i.e., a biomarker of nitrogen free radical species, NIT), and organic cation transporter 3 (i.e., a polyspecific transporter involved in the release of acetylcholine from non-neuronal cells, OCT3). Moreover, the impact of GPR55 activation on the level of vesicular acetylcholine transporter (VAChT) in the bladder detrusor muscle was also analyzed. All of these parameters may play a role in the functioning of the urinary bladder and/or in the pathophysiology of urological disorders. VAChT and OCT3 are relevant in the cholinergic transmission in the urinary bladder (Lips et al., 2007), CGRP may serve as a marker of bladder afferents involved in nociception (Dickson et al., 2006), whereas MAL and NIT could be indicators of the oxidative damage in the urinary bladder tissue (Gonzalez et al., 2015; Barut et al., 2019).

So far, there are no reports related to the possible role of the modulation of GPR55 receptors in the management of depression that coexists with bladder over activity. We hypothesize that stimulation of this atypical cannabinoid receptor may be a promising target in the treatment of the above-mentioned diseases. Thus, in the present study we investigated the effects of O-1602 in the corticosterone model of depression and detrusor overactivity in female Wistar rats.

## Materials and Methods

The experiments were carried out in accordance with European law related to the experimental studies on animal models and regulations on the ethical treatment of animals (Directive 2010/63/EU of the European Parliament and of the Council of 22). They were approved by the Local Ethics Committee in Lublin (No. 324/2019).

### Animals

60 female experimentally naïve Wistar rats (3 weeks old) with initial weight ranged between 200 and 225 g were used in the experiments (The Experimental Medicine Center of the Medical University of Lublin, Lublin, Poland). The animals were housed individually in metabolic cages which were placed in environmentally controlled rooms, with natural light/dark cycle, temperature of 22–23 °C, and relative humidity of 45–55%. Rats had free access to water and food, and they were subjected to the tests only once. 4 experimental groups consisted of 15 animals were tested in the experiments:

1^st^ group (CON) received vehicle for 14 days plus vehicle for 7 days
2^nd^ group (CORT) received corticosterone (20 mg/kg/day) for 14 days plus vehicle for 7 days 3^rd^ group (O-1602) received vehicle for 14 days plus O-1602 (0.25 mg/kg/day) for 7 days
4^th^ group (CORT + O-1602) received corticosterone (20 mg/kg/day) for 14 days plus O-1602 (0.25 mg/kg/day) for 7 days.

Repeated injections of corticosterone have been used for years as an animal model of depression in rats (Iijima et al., 2010) and mice (Zhao et al., 2008). We successfully refined this model in our lab (Wrobel et al., 2016; Wrobel et al., 2018).

### Drugs

For the first 14 days of the study, the tested rats were given subcutaneously (s.c.) corticosterone (Tocris Bioscience, UK). After that, a 7-day administration of O-1602 (Tocris Bioscence, UK) was introduced. O-1602 was dissolved in methyl acetate and administered intravenously into the right femoral vein through a polyethylene catheter. The control animals received a volume-matched injection of the vehicles (saline and/or methyl acetate). The doses of injected drugs as well as the pretreatment schedules were selected on the basis of the published data (Gratzke et al., 2009) and results of our previous studies (Wrobel et al., 2016; Wrobel et al., 2018).

### Surgical Procedures

The surgical procedures were carried out in the same way as had been previously described (Wrobel et al., 2016; Wrobel et al., 2018). The abdominal wall was opened with a vertical midline incision of approximately 10 mm. A double lumen catheter was inserted through the apex of the bladder dome and fixed with a 6-0 suture. In the same session the right femoral vein was catheterised. To prevent an infection of the urinary tract, 100 mg of cefazolin sodium hydrate (Biofazolin, Sandoz) was given s.c. All the surgical procedures were performed under anaesthesia with an intraperitoneal (i.p.) injection of ketamine hydrochloride (75 mg/kg; Ketanest, Pfizer) and xylazine (15 mg/kg; Sedazin, Biowet). Rats were laid down supine on a warm mattress (37 °C). A sufficient depth of anaesthesia was measured by lack of spontaneous movement and lack of withdrawal response to noxious toe pinch. According to the literature data (Cannon and Damaser, 2001), ketamine in combination with xylazine does not abolish the micturition reflex in female rats. We also have successfully used this popular method of anaesthesia for years [e.g., (Wrobel et al., 2015; Wrobel et al., 2016; Wrobel and Rechberger, 2017; Wrobel et al., 2018)].

### Conscious Cystometry

Cystometric measurements were carried out 3 days after the surgical procedures in the same way as had been previously described (Wrobel et al., 2015; Wrobel et al., 2016; Wrobel et al., 2018). The bladder catheter was connected *via* a three-way stopcock to a pressure transducer (FT03; Grass Instruments) and to a microinjection pump (CMA 100; Microject, Solna, Sweden). Cystometry was performed by slowly filling the bladder with physiological saline at a constant rate of 0.05 ml/min to elicit repetitive voiding. Micturition volumes were measured by means of a fluid collector attached to a force displacement transducer (FT03C; Grass Instruments). The measurements in each animal represent the average of five bladder micturition cycles after obtaining repetitive voiding. The following cystometric parameters were recorded: amplitude of nonvoiding contractions (ANVC; cm H_2_O), area under the pressure curve (AUC; cm H_2_O/sec), bladder compliance (BC; ml/cm H_2_O), basal pressure (BP; cm H_2_O), detrusor overactive index (DOI; cm H_2_O/ml), frequency of nonvoiding contractions (FNVC; times/filling phase), intercontraction interval (ICI; s), micturition voiding pressure (MVP; cm H_2_O), postvoid residual (PVR; ml), threshold pressure (TP; cm H_2_O), volume threshold to elicit nonvoiding contractions (VTNVC; %), and voided volume (VV; ml). The clinical meaning of the tested parameters was described previously (Wrobel et al., 2016).

### Behavioral Studies

The behavioral studies were performed 3 days after the last injection of O-1602. As the first one, the spontaneous locomotor activity was measured and then, the animals were subjected to the forced swim test (FST).

#### Locomotor Activity

The spontaneous locomotor activity was evaluated by an Optical Animal Activity Monitoring System (Digiscan apparatus; Omnitech Electronics, Columbus, OH, USA). At first, the animals were allowed to acclimatize to a new environment for 15 min and then, the standard 1-h analysis started. Horizontal activity, measured as interruptions of the infrared light beam in the horizontal sensor by a tested animal, was taken into consideration.

#### Forced Swim Test

The FST was carried out in the same way as we had described before (Wrobel et al., 2016; Wrobel et al., 2018). We applied a 15-min pre-test session, in which each rat was put individually into a dedicated glass cylinder (diameter 25 cm, height 65 cm) filled with water (temperature of 23–25°C). After 24 h, a 5-min standard session performed under identical conditions as the pre-test, took place. An animal was judged as immobile when it floated passively, performing only movements necessary to keep its head above the water level.

### Biochemical Analyses

After the cystometric measurements and behavioural tests, the rats were killed by decapitation and their urinary bladders and brains were collected. The following parameters were measured in the bladder urothelium: calcitonin gene related peptide (CGRP; Biomatik, CN EKU02858), malondialdehyde (MAL; Biomatik, CN EKF57996), 3-nitrotyrosine (NIT; Lifespan Biosciences; CN LS-F40120-1), and organic cation transporter 3 (OCT3; antibodies-online, CN ABIN6227163), whereas vesicular acetylcholine transporter (VAChT; Lifespan Biosciences, CN LS-F12924-1) level was assessed in the bladder detrusor muscle. Concentrations of interleukin 1-β (IL-1β; ELISA Kit for IL1b, Cloud-Clone, CNSEA563Ra), tumor necrosis factor alpha (TNF-α; ELISA Kit for TNF Alpha, Lifespan Biosciences, CN LS-F5193), corticotropin-releasing factor (CRF; Alpco, Salem, NH, USA, CN 48-CRFMS-E01), brain-derived neurotrophic factor (BDNF; PROMEGA, CN G7610), and nerve growth factor (NGF; LifeSpanBioSciences, CN LS-F25946-1) were evaluated in the hippocampus. Additionally, NGF and BDNF levels were determined in rats’ urine. Preparation of the samples as well as the measurements were performed according to the manufacturers’ instructions. Each sample was tested in duplicate, and the results were presented in pg/ml.

### Statistical Analysis

The statistical analysis was performed with two-way analysis of variance (ANOVA) (GraphPad Prism 6 Software). Bonferroni’s *post hoc* test was used, and p < 0.05 indicated a statistically significant difference between the tested groups. The obtained results were given as the means ± standard error of the mean (SEM).

## Results

### Forced Swim Test

**Figure 1:**
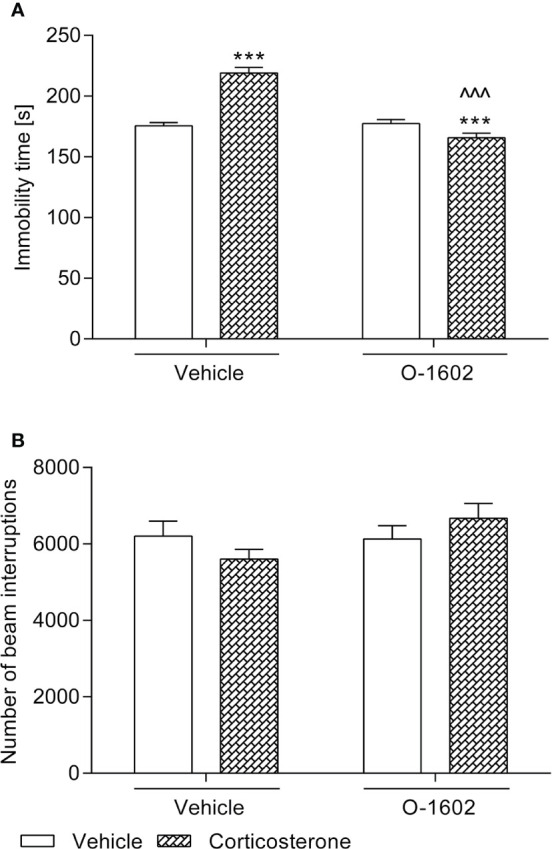
Influence of the 7-day intravenous administration of O-1602 (0.25 mg/kg/day) on the behaviour of rats subjected to the 14-day corticosterone treatment (20 mg/kg/day, subcutaneously) **(A)** in the forced swim test and **(B)** in the measurement of locomotor activity. The values represent the mean + SEM (n = 12 animals per group). ***p < 0.001 versus vehicle-treated group, ^^^p < 0.001 versus corticosterone-treated group (Bonferroni’s *post hoc* test).

A 14-day administration of corticosterone treatment (20 kg/mg/day) shortened the swimming time of the tested animals as compared to the vehicle-treated group. This effect was reversed by an intravenous 7-day therapy with O-1602 (0.25 mg/kg/day). Two-way ANOVA revealed a significant corticosterone-O-1602 interaction (F(1,44) = 55.52; p < 0.0001) with a significant effect of corticosterone (F(1,44) = 18.68; p < 0.0001) and a significant effect of O-1602 (F(1,44) = 47.76; p < 0.0001). Animals that received only vehicle and O-1602 behaved similarly to the control ones (**Figure 1A**).

### Locomotor Activity

As presented in **Figure 1B**, spontaneous locomotor activity of the animals was not disturbed by either of the tested substances.

### Cystometric Study

The 14-day corticosterone therapy (20 mg/kg/day) induced significant changes in several cystometric parameters, i.e. elevation of the ANVC, AUC, BP, DOI, FNVC values with reduction of the BC, ICI, TP, VTNVC, VV levels. PVR remained unaffected, whereas MVP was slightly lower than the control level (**Table 1**). The 7-day treatment with O-1602 (0.25 mg/kg/day) partially attenuated the noxious effects of the injected glucocorticoid, since it restored the AUC (two-way ANOVA: F(1,56) = 23.85; p < 0.0001 for the pretreatment-treatment interaction), BC (F(1,56) = 33.89; p < 0.0001), BP (F(1,56) = 10.01; p = 0.0025), ICI (F(1,56) = 5.21; p = 0.0263), VTNVC (F(1,56) = 19.46; p < 0.0001), and VV (F(1,56) = 14.73; p = 0.0003) to the control values. Moreover, administration of this GPR55 agonist moderately diminished the enhanced ANVC (two-way ANOVA: F(1,56) = 137.70; p < 0.0001 for the corticosterone- O-1602 interaction), DOI (F(1,56) = 91.55; p < 0.0001), and FNVC (F(1,56) = 91.84; p < 0.0001) levels. It also further reduced the MVP values, when given to the corticosterone-pretreated animals, and as a consequence, the observed differences *versus* the control group surpassed the significance level (**p < 0.01). Similarly, administration of O-1602 after corticosterone exposure further diminished the level of TP. Both the vehicle-pretreated and corticosterone-pretreated rats subjected to the O-1602 therapy presented considerably reduced PVRs.

**Table 1:** Influence of the 7-day intravenous administration of O-1602 (0.25 mg/kg/day) on the cystometric parameters in conscious rats subjected to corticosterone therapy.

Substances	amplitude of nonvoiding contractions [cm H_2_O]	area under the pressure curve [cm H_2_O/sec]	bladder compliance [ml/cm H_2_O]	basal pressure [cm H_2_O]	detrusor overactive index [cm H_2_O/ml]	frequency of nonvoiding contractions [times/filling phase]	intercontraction interval [s]	micturition voiding pressure [cm H_2_O]	postvoid residual [ml]	threshold pressure [cm H_2_O]	volume threshold to elicit nonvoiding contractions [%]	voided volume [ml]
vehicle (14 days) + vehicle (7 days)	2.540 ± 0.0809	20.27 ± 0.6864	0.3093 ± 0.0116	3.433 ± 0.1511	60.27 ± 3.073	0.8587 ± 0.0488	1065 ± 26.39	48.53 ± 1.316	0.075 ± 0.0023	8.907 ± 0.2579	73.93 ± 1.958	0.9713 ± 0.03747
CORT (14 days) + vehicle (7 days)	6.313 ± 0.1527***	31.20 ± 0.8685***	0.2420 ± 0.0125***	4.760 ± 0.3000***	364.6 ± 19.05***	7.240 ± 0.2903***	851.5 ± 27.59***	44.13 ± 1.983	0.069 ± 0.0033	6.647 ± 0.2855***	54.67 ± 2.142***	0.7953 ± 0.03217**
vehicle (14 days) + O-1602 (7 days)	2.613 ± 0.1125	18.07 ± 0.6208	0.2767 ± 0.0057	2.827 ± 0.1637	57.47 ± 3.830	1.041 ± 0.1032	1080 ± 27.43	52.20 ± 1.964	0.089 ± 0.0029*	9.127 ± 0.4546	66.87 ± 2.799	0.9347 ± 0.03066
CORT (14 days) + O-1602 (7 days)	3.327 ± 0.1596***^^^	22.07 ± 0.6360^^^	0.3360 ± 0.0123^^^	2.840 ± 0.1809^^^	140.5 ± 7.994***^^^	3.680 ± 0.2649***^^^	999.2 ± 34.14^^	40.13 ± 2.122**	0.090 ± 0.0046**^^^	4.900 ± 0.2719***^^^	67.87 ± 2.201^^^	1.045 ± 0.04691^^^

Corticosterone (CORT, 20 mg/kg/day) was given subcutaneously for 14 days and O-1602 (0.25 mg/kg/day) was administered intravenously for 7-days. The values represent the mean ± SEM (n = 15 rats per group). The obtained data were assessed by the two-way analysis of variance (ANOVA) followed by Bonferroni’s post hoc test. *p < 0.05, **p < 0.01, ***p < 0.001 versus saline, ^^p < 0.01, ^^^p < 0.001 versus CORT.

### Biochemical Study

The 7-day administration of O-1602 (0.25 mg/kg/day) in the vehicle-pretreated rats did not affect CGRP, MAL, NIT, OCT3, VAChT, IL-1β, TNF-α, CRF, BDNF, or NGF levels in the tested materials.

#### CGRP, MAL, NIT, OCT3 Levels in the Bladder Urothelium

**Figure 2:**
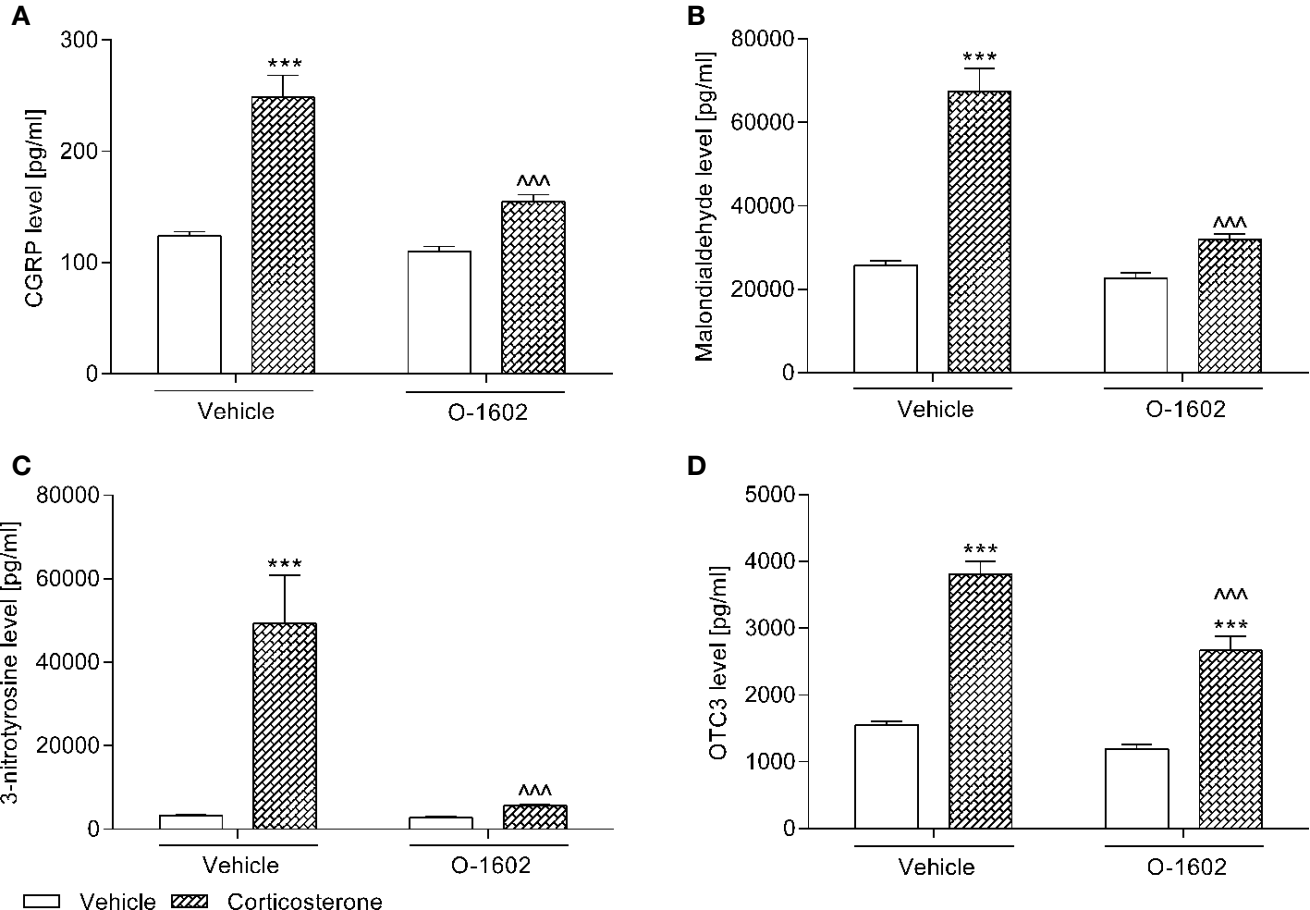
Influence of the 7-day intravenous administration of O-1602 (0.25 mg/kg/day) on the levels of **(A)** calcitonin gene related peptide (CGRP), **(B)** malondialdehyde, **(C)** 3-nitrotyrosine, and **(D)** organic cation transporter 3 (OCT3) in the the bladder urothelium of rats subjected to the 14-day corticosterone treatment (20 mg/kg/day, subcutaneously). The values represent the mean + SEM (n = 15 animals per group). ***p < 0.001 versus vehicle-treated group, ^^^p < 0.001 versus corticosterone-treated group (Bonferroni’s *post hoc* test).

After 14 days of corticosterone therapy (20 mg/kg/day) the tested parameters in the rats urothelium were significantly increased, i.e., the CGRP level by ca. 100% vs. the control group, MAL level by ca. 160%, NIT level by ca. 1100%, and OCT3 by ca. 145%. CGRP, MAL, and NIT values were normalized (i.e., restored to those of baseline controls) by the 7-day intravenous treatment with O-1602 (0.25 mg/kg/day), whereas administration of this GPR55 agonist only partially reduced the elevated concentration of OCT3. Two-way ANOVA detected a significant corticosterone-O-1602 interactions for the analysis of CGRP (F(1,56) = 13.67; p = 0.0005), MAL (F(1,56) = 30.27; p < 0.0001), NIT (F(1,56) = 13.77; p = 0.0005), and OCT3 (F(1,56) = 6.70; p = 0.0122). The results were presented in **Figure 2**.

#### VAChT Levels in the Bladder Detrusor Muscle

**Figure 3:**
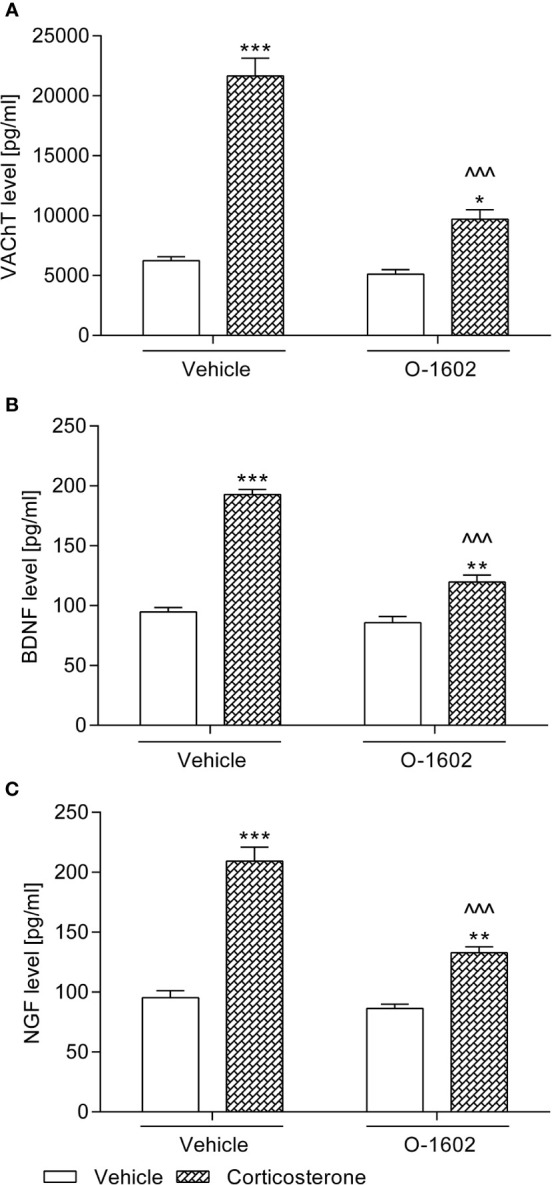
Influence of the 7-day intravenous administration of O-1602 (0.25 mg/kg/day) on the levels of **(A)** vesicular acetylcholine transporter (VAChT) in the bladder detrusor muscle, and **(B)** brain-derived neurotrophic factor (BDNF) with **(C)** nerve growth factor (NGF) in urine of rats subjected to the 14-day corticosterone treatment (20 mg/kg/day, subcutaneously). The values represent the mean + SEM (n = 15 animals per group). *p < 0.05, **p < 0.01, ***p < 0.001 versus vehicle-treated group, ^^^p < 0.001 versus corticosterone-treated group (Bonferroni’s *post hoc* test).

As illustrated in **Figure 3A**, rats subjected to the corticosterone treatment (20 mg/kg/day) for 14 days presented significantly increased VAChT levels in the bladder detrusor muscle (by ca. 250% vs. the vehicle-treated group). Though the 1-week therapy with O-1602 (0.25 mg/kg/day) considerably reduced the elevated VAChT values, they did not return to the basal level (*p < 0.05 vs. the control group). Two-way ANOVA showed a significant corticosterone-O-1602 interaction: F(1,56) = 37.85, p < 0.0001.

#### IL-1β, TNF-α, CRF, BDNF, and NGF Levels in the Hippocampus

**Figure 4:**
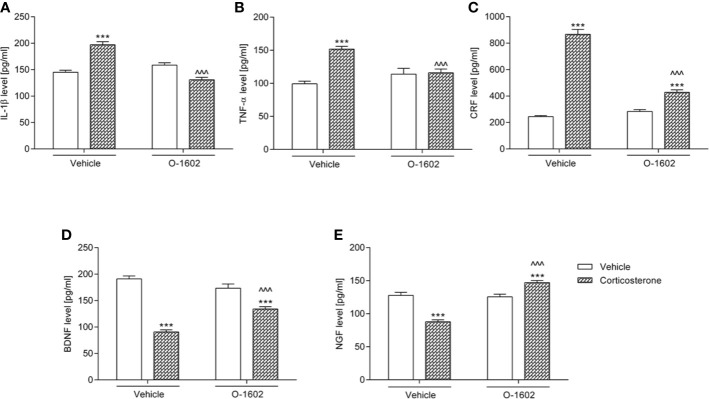
Influence of the 7-day intravenous administration of O-1602 (0.25 mg/kg/day) on the levels of **(A)** interleukin 1-β (IL-1β) **(B)** tumor necrosis factor alpha (TNF-α), **(C)** corticotropin-releasing factor (CRF), **(D)** brain-derived neurotrophic factor (BDNF), and **(E)** nerve growth factor (NGF) in the hippocampus of rats subjected to the 14-day corticosterone treatment (20 mg/kg/day, subcutaneously). The values represent the mean + SEM (n = 14-15 animals per group). ***p < 0.001 versus vehicle-treated group, ^^^p < 0.001 versus corticosterone-treated group (Bonferroni’s *post hoc* test).

**Figure 4** presents changes in the tested parameters after introduction of the corticosterone and/or O-1602 therapy. The 14-day exposition to glucocorticoid (given at a dose of 20 mg/kg/day) significantly elevated IL-1β (by ca. 35% vs. the vehicle-treated group), TNF-α (by ca. 53%), and CRF (by ca. 250%) levels in the hippocampus, whereas it diminished the concentration of BDNF (by ca. 52%) and NGF (by ca. 31%). O-1602 when given for 7 days at a dose of 0.25 mg/kg/day managed to reverse the noxious effects of corticosterone – it normalized the hippocampal values of IL-1β, TNF-α, and NGF, as well as it partially reduced the elevated levels of CRF and raised the lowered concentrations of BDNF. Two-way ANOVA revealed a significant pretreatment-treatment interactions: F(1,56) = 71.60; p < 0.0001 for the analysis of IL-1β, F(1,56) = 18.03; p < 0.0001 for the analysis of TNF-α, F(1,56) = 114.45; p < 0.0001 for the analysis of CRF, F(1,55) = 29.34; p < 0.0001 for the analysis of BDNF, and F(1,56) = 65.90; p < 0.0001 for the analysis of NGF.

#### BDNF and NGF Levels in Urine

Subcutaneous injections of corticosterone at a dose of 20 mg/kg/day for 2 weeks induced a considerable increase of BDNF and NGF levels in urine of the tested rats (by ca. 100% and 120%, respectively vs. the control group). After introduction of the O-1602 therapy (0.25 mg/kg/day for 7 days), urine secretion of both BDNF and NGF was significantly reduced, though it did not return to the basal values (**Figures 3B, C**). The following outcomes of the statistical analysis (two-way ANOVA) regarding the corticosterone-O-1602 interaction were obtained in the case of the BDNF level: F(1,56) = 44.85; p < 0.0001 and in the case of the NGF level: F(1,56) = 21.62; p < 0.0001.

## Discussion

Though it should be admitted that both genetically-induced and pharmacologically-induced disturbances in the signaling dependent on CB_1_ and CB_2_ receptors may lead to depressive phenotype, modulation of the endocannabinoid system can have an antidepressant-like potential as well. Interestingly, in multiple pre-clinical studies, agonists and antagonists/inverse agonists of CB_1_ and CB_2_ receptors exerted antidepressant effects in rodents. Moreover, we previously demonstrated that they managed to enhance the activity of conventional antidepressant drugs belonging to different pharmacological classes, such as imipramine, escitalopram, and reboxetine (Poleszak et al., 2019). On the other hand, mice and rats with experimentally-induced depression as well as depressed patients and suicide victims present diverse abnormalities in the endocannabinoid system [for review see (Poleszak et al., 2018)]. Similarly, it has been demonstrated that CB_1_ receptors are significantly up-regulated in the urothelium and down-regulated in the detrusor of patients with detrusor overactivity (Leron et al., 2018). According to the experiments by Hiragata et al. (2007), a synthetic analog of tetrahydrocannabinol suppressed both normal bladder activity and urinary frequency in cyclophosphamide-treated rats. However, available literature does not provide enough data to confirm whether atypical cannabinoids that bind to GPR55 receptors retain properties of the traditional cannabinoid ligands against depression and detrusor overactivity. Nevertheless, such an analogy has been demonstrated for the anxiolytic-like effects. Even if mice with GPR55 receptors knock-out behaved similarly to the wild-type animals in the elevated plus maze (Wu et al., 2013), activation of GPR55 receptors was anxiolytic, whereas their antagonism was anxiogenic (Rahimi et al., 2015). Moreover, the early-life stress induced changes in the expression of the GPR55 gene in mice (Marco et al., 2014). So far it is also known that GPR55 receptors have a certain role in modulating emotional states and that they closely co-operate with CB_2_ receptors. García-Gutiérrez and colleagues (2018) detected the reduced expression of the GPR55 gene along with enhanced expression of CB_2_-GPR55 heteromers in the dorsolateral prefrontal cortex of suicide victims.

To the best of our knowledge this is the first report of the positive effects of O-1602 in an animal model of depression that co-exists with detrusor overactivity. O-1602 is a phytocannabinoid-like analogue of cannabidiol which similarly to other atypical cannabinoids does not bind to CB_1_ or CB_2_ receptors. It is an agonist of GPR55 receptors with a wide biological activity demonstrated in pre-clinical studies. Additionally, this compound is also known as a biased agonist of the GPR18 receptors (Console-Bram et al., 2014). O-1602 possesses vasodilatory and hypotensive properties (Johns et al., 2007), enhances insulin release from pancreatic cells (Liu et al., 2016), and increases feeding in rats (Diaz-Arteaga et al., 2012). Besides, it induced the anti-inflammatory and pain relieving effects in diverse animal models of inflammation (Schuelert and McDougall, 2011; Schicho et al., 2011; Li et al., 2013) and had a pronociceptive activity in neuropathic pain (Breen et al., 2012). Several authors noted that administration of O-1602 to both normal (Rahimi et al., 2015) and stressed (Shi et al., 2017) rodents resulted in the anxiolytic-like activity and this effect was mediated *via* GPR55 receptors since it was abolished by GPR55 antagonists (i.e., CID16020046 or ML-193) and GPR55 receptor knock-down (Rahimi et al., 2015; Shi et al., 2017). In the presented study, we found that O-1602 has an antidepressant-like potential and the potential against detrusor overactivity. The tested substance at an intravenous dose of 0.25 mg/kg/day given for 7 days reversed the noxious effects of a 14-day exposure to corticosterone treatment (20 mg/kg/day). It reversed the significantly prolonged immobility time of rats in the FST indicating the depressive phenotype and the undesired changes in cystometric parameters demonstrating the overactive detrusor. Since there are no available data on the potential activity of O-1602 in depression co-existing with detrusor overactivity/overactive bladder, when selecting the tested dose, we decided to base it on the report of Gratzke et al. (2009). The preliminary (unpublished) experiments carried out in our lab revealed that the lowest active intravenous dose of O-1602 in the conditions of our study was 0.25 mg/kg. The treatment duration (7-days) was chosen on the basis of the outcomes of our previous project (Wrobel et al., 2018), in which another experimental substance, GSK 269962 (an inhibitor of Rho kinase) reversed both corticosterone-induced detrusor overactivity and depression-like behaviour in rats. It should be noted that though Alavi et al. (2016) reported enhanced locomotor activity of animals after administration of O-1602 (5 mg/kg, intraperitoneally) caused by a possible influence on the dopaminergic neurotransmission, we did not detect any disturbances in the spontaneous activity of the tested rats. Our results were in line with the outcomes of Schicho et al. (2011) and Rahimi et al. (2015). Thus, we can assume that the observations made in the FST were not confounded by either hypo- or hyperlocomotion potentially induced by the introduced treatment.

The cystometric analysis revealed that O-1602 therapy restored the elevated AUC and BP levels as well as diminished BC, ICI, VTNVC, and VV levels to the control values. Stimulation of GPR55 receptors also moderately reduced the increased ANVC, DOI, and FNVC levels. Decrease of DOI values is particularly satisfactory since this parameter seems to describe the grade of detrusor overactivity more precisely than the others (Wrobel and Rechberger, 2017). It should be mentioned that detrusor overactivity is a frequent symptom of overactive bladder – 64% of patients with overactive bladder have overactive detrusor and 83% of patients with detrusor overactivity is diagnosed with overactive bladder (Abrams and Andersson, 2007). It turned out that O-1602 reduced MVP values in the corticosterone-subjected rats, though such an effect was not recorded in the vehicle-pretreated animals. Surprisingly, therapy with the tested atypical cannabinoid significantly elevated PVR levels. Decrease in MVP accompanied by increase in PVR may suggest that O-1602 at the applied dose exerts its influence not only on the urine storage phase, but also on voiding. It seems to be a result of O-1602-induced reduction of VAChT levels in the bladder detrusor.

Since a number of pre-clinical and clinical studies has confirmed a role of the hypothalamic-pituitary-adrenal axis, neurotrophins, and immune activation in the pathogenesis of depression and detrusor overactivity, we wanted to know whether activation of GPR55 receptors is able to abolish the corticosterone-induced disturbances in the urine and/or the hippocampus levels of IL-1β, TNF-α, CRF, BDNF, and NGF in female rats. The outcomes of our experiments generally confirmed that the prolonged exposure to glucocorticoids exerts the detrimental neuronal effects as well as enhances the peripheral and central production of pro-inflammatory cytokines. Correspondingly to the trend observed in different tissue samples collected from depressed people (Felger and Lotrich, 2013), elevated levels of pro-inflammatory cytokines (i.e., IL-1β, TNF-α) and CRF with decreased values of BDNF and NGF were detected in the hippocampus of corticosterone-subjected rats. Moreover, pretreatment with this drug induced a significant augmentation of BDNF and NGF concentrations in urine. Elevated urinary NGF and BDNF levels are usually observed in patients with detrusor overactivity (Frias et al., 2011). According to the literature data (Leron et al., 2018), it cannot be ruled out that neurotrophic factors are at least partially responsible for the altered expression of CB_1_ receptors in patients with detrusor overactivity. Although the introduced O-1602 therapy was not potent enough to normalize the hippocampal and urine levels of the tested biomarkers, it managed at least partially to reverse all of the identified biochemical changes. In fact, both involvement of GPR55 receptors in inflammation (including neuroinflammation) and anti-TNF-α properties of the O-1602 intraperitoneal therapy (at a dose of 10 mg/kg given twice) had been reported before (Li et al., 2013). According to available data, effects of the GPR55 receptor stimulation in the inflammatory processes may be bi-directional, i.e. pro-inflammatory (Chiurchiu et al., 2015) or anti-inflammatory (Hill et al., 2019), depending on the study design and experimental conditions. In the *in vitro* studies by Chiurchiu et al. (2015), O-1602 enhanced production of IL-12 and TNF-α in lipopolysaccharide-activated monocytes and negatively affected its ability to phagocytose, whereas in the *in vitro* studies by Hill et al. (2019), exposure to O-1602 suppressed pro-inflammatory responses within neural stem cells during their insult with IL-1β. Furthermore, animals genetically deprived of GPR55 receptors presented prolonged inflammatory response (with elevated IL-1β, IL-6, TNF-α values) after chronic low-level administration of lipopolysaccharide (Hill et al., 2019).

Biochemical analysis carried out in the presented study further showed that the pro-inflammatory response and disturbances in the cystometric parameters induced by administration of corticosterone at a dose of 20 mg/kg/day for 14 days were accompanied by the oxidative damage in the bladder urothelium, which was detected by the elevated values of MAL and NIT. MAL and NIT belong to recognized markers of the cellular oxidative damage and their increased levels had been recorded in bladder disorders manifested by detrusor overactivity and bladder inflammation (Gonzalez et al., 2015; Barut et al., 2019). Moreover, it turned out that the cystometric changes detected after corticosterone exposure may be due to an increase in the activity of afferent fibers located in the urothelium and due to enhanced transport of acetylcholine to the presynaptic vesicles. The corticosterone-subjected rats had significantly elevated levels of CGRP and OCT3 in the bladder urothelium and VAChT levels in the bladder detrusor muscle. Our outcomes are in line with the experiments by Dickson and colleagues (Dickson et al., 2006) who found an overall increase in the density of CGRP- and VAChT-immunoreactive fibers in the urinary bladder mucosa of rats with cyclophosphamide-induced cystitis. High concentrations of CGRP were also detected in samples taken from bladders of female patients suffering from this disease (Smet et al., 1997). Furthermore, botulinum toxin A (i.e., a substance known for relieving symptoms of detrusor overactivity) as well as cannabinoid agonists reduced CGRP release from afferent terminals (Rapp et al., 2006; Hayn et al., 2008). Both centrally and peripherally distributed CGRP is implicated in nociception and inflammatory responses (Dickson et al., 2006). The membrane protein VAChT is responsible for transporting acetylcholine (i.e., the most important transmitter in voiding and urinary bladder contraction) from cytoplasm to presynaptic vesicles, thereby influencing its release. The polyspecific transporter OCT3, which is also present in human urothelium, is capable of translocating acetylcholine across the plasma membrane, contributing to acetylcholine release from the bladder mucosa (Lips et al., 2007). The results of our experiments revealed that the O-1602 treatment at least partially restored the proper functioning of the tested urinary bladders of animals presenting the signs of detrusor overactivity. It successfully reversed all of the above-mentioned biochemical alterations in the bladder urothelium and detrusor muscle. Only OCT3 and VAChT values did not drop enough to reach the control levels, however they became significantly reduced.

We have to admit that at the moment we are not able to describe the exact molecular mechanism of the observed effects of O-1602. We can only assume that its antidepressant-like activity is most probably due to the potent agonism of GPR55 receptors, as it has been confirmed for its anxiolytic effects. Shi and colleagues (2017) suggested that in the emotional-modulating effects of O-1602, the RhoA/RhoA kinase and phospholipase C/protein kinase C signaling along with the extracellular-signal-regulated kinase pathway may be involved. According to the above-mentioned authors (Shi et al., 2017), other transmissions affected by GPR55 receptors, e.g., p38-, NFAT- or Rac-dependent ones, could contribute to the observed results as well. Kargl et al. (2013) noted that O-1602 also influences STAT3- and NFκB p65-dependent pathways; both of them are implicated in the activity of the urinary bladder. Additionally, in the *in vitro* experiments by Lin et al. (2011), O-1602 was able to slow down spontaneous and stimulated contractions of the intestinal smooth muscle. Alternatively, based on the studies by Schicho et al. (2011) and Johns et al. (2007), transmission dependent on other (than GPR55) atypical receptors (such as the transient receptor potential vanilloid-1 – TRPV1 (Schuelert and McDougall, 2011) or the orphaned receptor GPR18, both probably interacting with O-1602 (McHugh et al., 2010) could also be partially responsible for the obtained results. Participation of the GPR18 receptor-dependent pathways should be particularly expected since Reyes-Resina et al. (2018) revealed their role in neurodegenerative processes, whereas Liu and colleagues (2019) demonstrated that the GPR18 gene belongs to the key genes in ulcerative interstitial cystitis/pain bladder syndrome.

We think that the main limitation of the current study is a lack of experiments assessing the potential changes in the central nervous system induced by the treatment with O-1602. The presented work was focused on the behavioural aspects and biochemical alterations in the peripheral system. However, additional tests measuring for example the expression of receptors and levels of neurotransmitters involved in the development of depression and/or micturition process would enable better understanding of the molecular mechanisms implicated in our findings.

## Conclusion

To the best of our knowledge, this is the first study showing that O-1602 therapy improves signs of depression and detrusor contractility as well as it ameliorates the oxidative and inflammatory damage in female rats exposed to corticosterone treatment. Accordingly, the following outcomes of the presented experiments should be particularly emphasized: (1) corticosterone-induced depression and detrusor overactivity is accompanied by signs of inflammation, disturbances in concentrations of neurotrophic factors in urine and/or the hippocampus, oxidative damage in the urinary bladder tissue, as well as by abnormalities in the levels of the calcitonin gene related peptide, organic cation transporter 3, and vesicular acetylcholine transporter in the bladder detrusor muscle or bladder urothelium, (2) agonism of the orphan metabotropic receptor GPR55 reverses the symptoms of depression and detrusor overactivity in female rats exposed to corticosterone treatment, (3) agonism of the orphan metabotropic receptor GPR55 reverses the changes in several biomarkers associated with depression and/or detrusor overactivity in the hippocampus, bladder urothelium, bladder detrusor muscle, and/or urine in female rats exposed to corticosterone treatment.

The above-mentioned findings allow to suggest that the potential clinical use of the modulation of atypical cannabinoid receptors GPR55 could be widened with the possible role in the treatment of depression and overactive bladder. However, further confirmatory studies are needed.

## Data Availability Statement

The raw data supporting the conclusions of this article will be made available by the authors, without undue reservation.

## Ethics Statement

The animal study was reviewed and approved by the Local Ethics Committee in Lublin.

## Author Contributions

AW and TR conceived and designed the research. AW, ASz, EP, and DU conducted experiments. AW and ASe analyzed the data. AW and ASe interpreted the results. ASe wrote the manuscript. All authors contributed to the article and approved the submitted version.

## Funding

This study was supported by Funds for Statutory Activity of Medical University of Lublin, Poland.

## Conflict of Interest

The authors declare that the research was conducted in the absence of any commercial or financial relationships that could be construed as a potential conflict of interest.

## Abbreviations

3-NIT, 3-nitrotyrosine; ANVC; amplitude of nonvoiding contractions; AUC, area under the pressure curve; BP, basal pressure; BC, bladder compliance; BDNF, brain-derived neurotrophic factor; CGRP, calcitonin gene related peptide; CREB, cAMP response element binding; CB receptor, cannabinoid receptor; CRF, corticotropin-releasing factor; DO, detrusor overactivity; DOI, detrusor overactive index; FST, forced swim test; FNVC, frequency of nonvoiding contractions; ICI, intercontraction interval; IL-1β, interleukin 1β; MAL, malondialdehyde; MVP, micturition voiding pressure; NGF, nerve growth factor; NF-κB, nuclear factor kappa-light-chain-enhancer of activated B cells; NFAT, nuclear factor of activated T-cells; OCT3, organic cation transporter 3; PVR, postvoid residual; SEM, standard error of the mean; TP, threshold pressure; TNF-α, tumor necrosis factor α; VAChT, vesicular acetylcholine transporter; VV, voided volume; VTNVC, volume threshold to elicit nonvoiding contractions.
